# P-329. Leveraging Hospital-Community Partnerships to Support Equitable Access and Adherence to Long-acting Injectable PrEP (LAI-PrEP) at a NYC Safety-Net Hospital: Findings from the EquiPrEP Study

**DOI:** 10.1093/ofid/ofaf695.548

**Published:** 2026-01-11

**Authors:** Robert Pitts, Ofole Mgbako, Katharine Ramos, Judith Ratcliffe, Emma Kaplan-Lewis, Eunice Casey, Farzana Kapadia, Brandi Moore, Sahnah Lim, Rosa Shapiro-Thompson

**Affiliations:** NYU Langone Health, New York, New York; NYC Health+Hospitals, Brooklyn, NY; Health & Hospitals/Bellevue, New York, New York; NYC Health+Hospitals, Brooklyn, NY; NYC Health and Hospitals, NY, New York; NYC Health and Hospitals, NY, New York; NYU School of Global Public Health, New York, New York; New York University School of Global Public Health, New York, New York; NYU Grossman School of Medicine, New York, New York; NYU Grossman School of Medicine, New York, New York

## Abstract

**Background:**

LAI-PrEP requires innovative strategies to increase access to underserved communities and support adherence. EquiPrEP is an implementation science study launched at a NYC municipal hospital in partnership with 4 community-based organizations (CBOs). We developed collaboratively a bi-directional referral process to link CBO clients to LAI-PrEP and to connect patients to CBOs for health-related social needs. We describe the CBO/clinic referral model, 6-month adherence for patients referred from CBOs, and results from interviews with CBO staff to evaluate this partnership.

**Figure:**
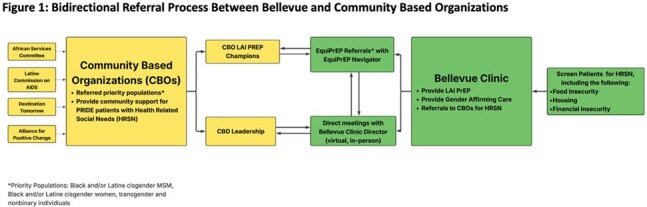

**Figure:**
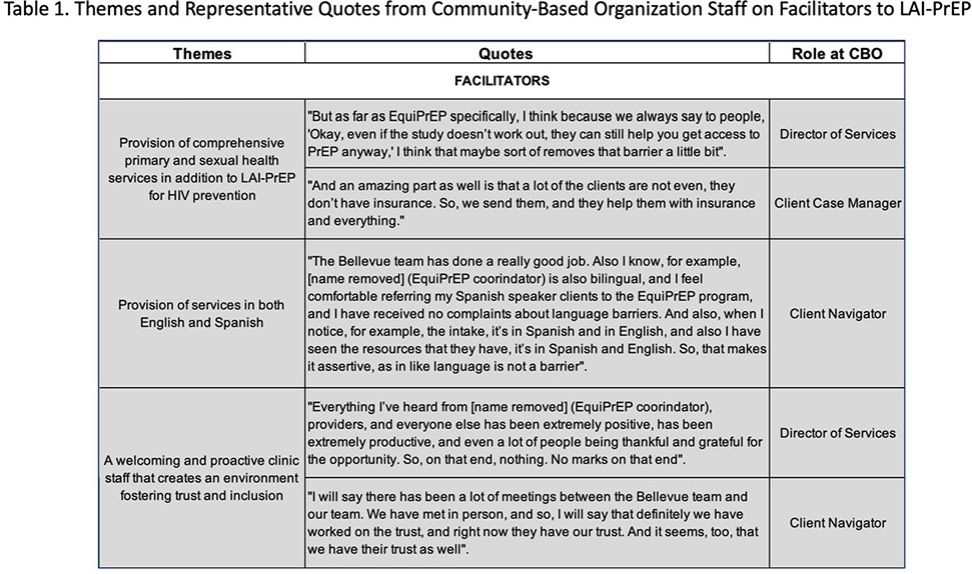

**Methods:**

From 02/2023 – 12/2024, referral source, enrollment status, and adherence at 6-months (defined as on-time injections +/- 7 days) were collected among participants at baseline and follow up. In-depth interviews were conducted among 9 CBO staff (case managers, navigators, and director of client services) involved in referral processes and analyzed by 2 qualitative experts for barriers and facilitators to LAI-PrEP access and adherence.

**Figure:**
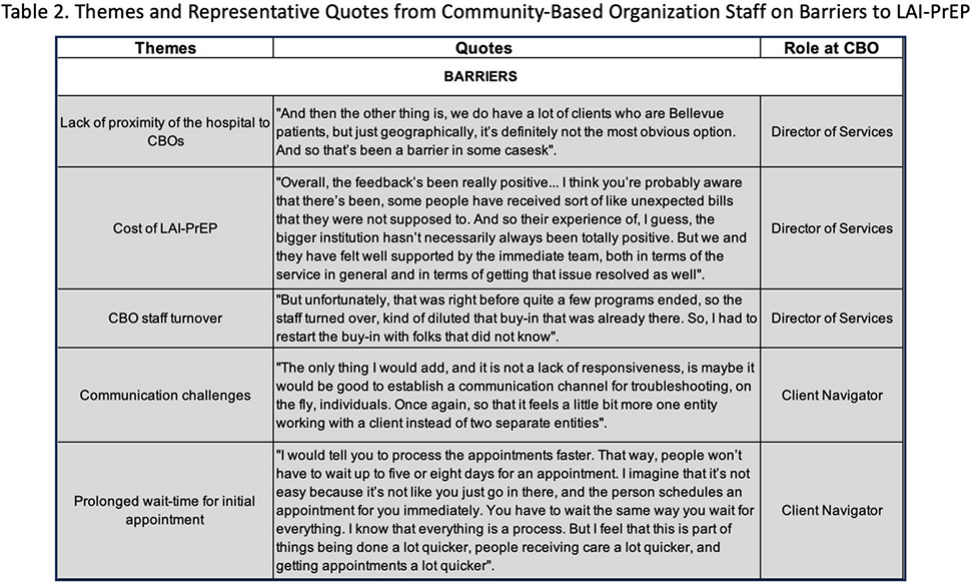

**Results:**

Figure 1 illustrates the bidirectional referral process. CBOs referred 28 individuals, 61% (n=17) enrolled into EquiPrEP and initiated LAI-PrEP, the 11 individuals referred but not enrolled were offered routine clinical care. Of those enrolled, 71% (n=12) were fully adherent to LAI-PrEP at 6-months. CBO staff identified the following factors as key to facilitating LAI-PrEP initiation and adherence: (1) provision of comprehensive primary and sexual health services in addition to LAI-PrEP for HIV prevention, (2) provision of services in Spanish, and (3) a welcoming and proactive clinic staff that creates an environment fostering trust and inclusion. Major barriers identified by CBO staff include: (1) lack of proximity of the hospital to CBOs, (2) cost of LAI-PrEP, (3) CBO staff turnover, (4) communication challenges, and (5) prolonged wait-time for initial appointment (Table 1 & 2).

**Conclusion:**

The support to patients/clients is strengthened by availability of bi-directional referrals to clinical and support services through a coordinated, mutually beneficial partnership. Healthcare systems should continue to invest resources to improve the ability for CBOs and clinics to collaborate and support HIV prevention as part of comprehensive sexual health care.

**Disclosures:**

Robert Pitts, MD MPH, Gilead Inc: Advisor/Consultant|ViiV: Advisor/Consultant Ofole Mgbako, MD, MS, Gilead Sciences: Advisor/Consultant Emma Kaplan-Lewis, MD, gilead: Grant/Research Support

